# Non-Invasive Assessment of Hepatic Steatosis in Patients with NAFLD Using Controlled Attenuation Parameter and ^1^H-MR Spectroscopy

**DOI:** 10.1371/journal.pone.0091987

**Published:** 2014-03-17

**Authors:** Thomas Karlas, David Petroff, Nikita Garnov, Stephan Böhm, Hannelore Tenckhoff, Christian Wittekind, Manfred Wiese, Ingolf Schiefke, Nicolas Linder, Alexander Schaudinn, Harald Busse, Thomas Kahn, Joachim Mössner, Thomas Berg, Michael Tröltzsch, Volker Keim, Johannes Wiegand

**Affiliations:** 1 IFB AdiposityDiseases, Leipzig University Medical Center, Leipzig, Germany; 2 Department of Medicine, Neurology and Dermatology, Division of Gastroenterology and Rheumatology, University Hospital Leipzig, Leipzig, Germany; 3 Clinical Trial Center, University of Leipzig, Leipzig, Germany; 4 Department of Diagnostics and Interventional Radiology, University Hospital Leipzig, Leipzig, Germany; 5 Department of Medicine, Neurology and Dermatology, Division of Gastroenterology and Rheumatology, Section of Hepatology, University Hospital Leipzig, Leipzig, Germany; 6 Institute of Pathology, University Hospital Leipzig, Leipzig, Germany; 7 Clinic for Gastroenterology and Hepatology, Klinikum St. Georg, Leipzig, Germany; Lady Davis Institute for Medical Research/McGill University, Canada

## Abstract

**Introduction:**

Non-invasive assessment of steatosis and fibrosis is of growing relevance in non-alcoholic fatty liver disease (NAFLD). ^1^H-Magnetic resonance spectroscopy (^1^H-MRS) and the ultrasound-based controlled attenuation parameter (CAP) correlate with biopsy proven steatosis, but have not been correlated with each other so far. We therefore performed a head-to-head comparison between both methods.

**Methods:**

Fifty patients with biopsy-proven NAFLD and 15 healthy volunteers were evaluated with ^1^H-MRS and transient elastography (TE) including CAP. Steatosis was defined according to the percentage of affected hepatocytes: S1 5-33%, S2 34–66%, S3 ≥67%.

**Results:**

Steatosis grade in patients with NAFLD was S1 36%, S2 40% and S3 24%. CAP and ^1^H-MRS significantly correlated with histopathology and showed comparable accuracy for the detection of hepatic steatosis: areas under the receiver-operating characteristics curves were 0.93 vs. 0.88 for steatosis ≥S1 and 0.94 vs. 0.88 for ≥S2, respectively. Boot-strapping analysis revealed a CAP cut-off of 300 dB/m for detection of S2-3 steatosis, while retaining the lower cut-off of 215 dB/m for the definition of healthy individuals. Direct comparison between CAP and ^1^H-MRS revealed only modest correlation (total cohort: r = 0.63 [0.44, 0.76]; NAFLD cases: r = 0.56 [0.32, 0.74]). For detection of F2–4 fibrosis TE had sensitivity and specificity of 100% and 98.1% at a cut-off value of 8.85 kPa.

**Conclusion:**

Our data suggest a comparable diagnostic value of CAP and ^1^H-MRS for hepatic steatosis quantification. Combined with the simultaneous TE fibrosis assessment, CAP represents an efficient method for non-invasive characterization of NAFLD. Limited correlation between CAP and ^1^H-MRS may be explained by different technical aspects, anthropometry, and presence of advanced liver fibrosis.

## Introduction

Hepatic steatosis is commonly observed upon histopathological evaluation of patients with different chronic liver diseases like alcoholic or non-alcoholic fatty liver disease (NAFLD), chronic hepatitis B or C or drug induced liver injuries (e.g. by long-term corticosteroid or amiodarone exposure) [1], [2]. NAFLD is the most prevalent chronic liver disease in the Western world and affects up to 30% of the population [3]. Its spectrum ranges from simple steatosis and steatohepatitis to fibrosis and long-term complications like liver cirrhosis and hepatocellular carcinoma [4], [5]. The reliable quantification of hepatic steatosis is of growing clinical relevance, because increasing steatosis may favor progression of fibrosis [3], [6] and limit treatment response, e.g. in patients with viral hepatitis [7], [8]. Moreover, detailed quantification of steatosis is important in estimating the therapeutic success of different pharmaceutical treatment options in NAFLD [9], [10].

The current gold standard for the assessment of hepatic steatosis and associated necroinflammatory activity is liver biopsy. However, results can be limited by sampling errors, intra- and inter-observer variability and difficulties in acquiring repetitive and longitudinal data due to the invasiveness of the procedure [11], [12]. Therefore, a number of imaging or laboratory based methods (e.g. SteatoTest [13], Fatty Liver Index [14]) have been developed in the last years to quantify hepatic steatosis non-invasively. Imaging modalities include ultrasound, computed tomography (CT), and magnetic resonance imaging (MRI) [15]. Recently, an additional ultrasound based method named Controlled Attenuation Parameter (CAP) has been developed to investigate hepatic steatosis [16]. CAP is included in the transient elastography system (TE), which uses vibration induced elastic shear-waves for assessment of liver stiffness. For the 3.5 MHz TE M probe, the CAP algorithm calculates the attenuation of ultrasonic signals used for characterization of the shear-wave propagation. In contrast to conventional B-mode ultrasound, which is impaired by low sensitivity and difficulties in differentiating different grades of hepatic steatosis, CAP has shown adequate performance for the detection and semi-quantification of steatosis in several biopsy-controlled clinical studies [17]–[22].

1H-magnetic res°nance spectroscopy (^1^H-MRS) is a safe and non-invasive alternative for quantification of hepatic fat content which offers good reproducibility and detailed investigation of different liver lobes and has been studied in various clinical studies [23]–[26]. Although potential limitations are high costs and availability of the method, it seems especially helpful in clinical scenarios in which quantitative data of hepatic steatosis are of relevance [15].

Both the CAP technology and ^1^H-MRS reliably estimate the hepatic fat content and correlate with the histopathological evaluation of hepatic steatosis [27], [28]. However, a direct comparison between both non-invasive methods has not been performed yet. These data can be important to determine which non-invasive imaging method may be used in future clinical studies to investigate the clinical course of patients with NAFLD. We therefore performed a cross-sectional trial with head-to-head comparison between CAP and ^1^H-MRS.

## Patients and Methods

### Patients and controls

Between March and December 2012 outpatients with biopsy-proven NAFLD or NASH and absence of concomitant liver diseases were invited to participate in the study. Healthy volunteers without any known liver disease, diabetes mellitus, or metabolic syndrome were enrolled as control group. Weekly alcohol consumption above 210 g for men and 140 g for woman was ruled out for all study participants by a thorough clinical interview prior to inclusion [1]. In healthy controls, signs of hepatic steatosis in conventional ultrasound (increased echogenicity pattern of liver parenchyma compared to the right renal cortex using a conventional convex ultrasound probe) were regarded as exclusion criterion.

All patients underwent clinical examination, abdominal ultrasound, liver stiffness measurement with transient elastography combined with measurement of controlled attenuation parameter and laboratory assessment on the same day of presentation. In addition, ^1^H-MRS was performed within a time period of three weeks, in the majority of cases on the day of ultrasound assessment. Fasting for at least three hours was required prior to the ultrasound and elastography examinations.

### Ethics statement

The study was performed in accordance with the ethical guidelines of the Helsinki Declaration and was approved by the local ethics committee (University of Leipzig, register no. 283-11-22082011). All participants provided written informed consent.

### Liver histology

Diagnosis of NAFLD or NASH was based on liver biopsy. The NAFLD activity score (NAS) was assessed by a single expert pathologist blinded to the clinical data. Steatosis was defined according to the number of affected hepatocytes: S1 (5–33%, “mild”), S2 (33–66%, “moderate”), S3 (>66%, “severe”). Fibrosis was classified according the NAS staging [1], [29].

A time interval between liver biopsy and study inclusion of up to 48 months was arbitrary accepted for enrolment in the study (median interval between biopsy and study inclusion 8.5 months, range 0–40 months).

### Elastography, CAP, and ultrasound

All subjects underwent liver stiffness measurement using the M probe of transient elastography (Echosens, Paris, France; Software Version 2.01.4_1889). In brief, the device transmits a mechanical vibration to the tissue and induces elastical shear-wave propagation which is tracked by pulse-echo ultrasound signals at a measuring depth of 2.5 to 6.5 cm [30]. The shear-wave velocity is directly related to the tissue stiffness and expressed in kPa. TE was performed in supine position in a right intercostal space. Ten valid measurements were taken with the M probe according to the manufacturer's recommendation. A success rate of >60% was required for a valid measurement. Examinations with an interquartile range (IQR) >30% of the median liver stiffness value were classified as unreliable and excluded from further analysis [31]. The controlled attenuation parameter (CAP) represents the ultrasonic attenuation coefficient of the ultrasonic signals used during the TE examination and is expressed in dB/m. The technical background has been recently described in detail [16]. The algorithm is included in the TE software and data are automatically calculated simultaneously with the liver stiffness measurement. CAP was only appraised in case of a valid and reliable TE measurement [16], [19].

The distance between skin and liver capsule at the site of TE measurement was measured using a conventional linear ultrasound transducer.

### Magnetic resonance spectroscopy and volumetry

MR examinations were performed on a 1.5-T scanner (Achieva XR, Philips Healthcare, Best, Netherlands) with the patient in supine position. Single-voxel MR spectra were acquired with the integrated body coil using a point-resolved spectroscopy (PRESS) technique and local shimming. Voxels (size 20×20×20 mm^3^) were placed in the right liver lobe (segment VII) trying to avoid bile ducts and larger vessels. Scans were acquired during free breathing (using breath triggering) with the following sequence parameters: repetition time, TR = 3,500 ms, echo time, TE = 25 ms, 512 data points, bandwidth, BW = 1,000 Hz/pixel, 40 averages, total acquisition time, TA = 140 s, and without water suppression.

MR spectra were analyzed with a commercial tool that uses an optimized set of basis functions to determine the relative concentrations of hepatic lipids (LCModel 6.3, Oakville, Canada). Calculated peak areas of water and fat were corrected for T2 relaxation applying previously published literature values [32] and were used to calculate the liver fat content (hepatic fat fraction; given in %) according to the ratio LFC  =  Sfat/(Sfat + Swater) with Sfat as the sum of the areas under the methyl (0.9 ppm), methylene (1.3 ppm) and allylic (2.1 ppm) peaks and Swater as the area under the water peak (4.7 ppm).

The liver volume calculation was performed using a custom-made software tool (Matlab, MathWorks, Natick, MA, USA). The liver was manually segmented on the images acquired with an in-phase/opposed-phase sequence described in Thörmer et al. 2013 [33] trying to avoid bile ducts and larger vessels. The volume of the visceral adipose tissue (VAT) and subcutaneous adipose tissue (SAT) was determined using software for automated abdominal fat quantification [34] in a single slice (10 mm thick) at the level of the lower end of L3 which represents the best association to the abdominal fat volume [34]–[36].

### Laboratory assessment, NAFLD fibrosis score, and PNPLA3 genotyping

Blood samples were collected from all study participants after the ultrasound examinations. Blood count and serum levels of aminotransferases (ALT and AST), glycohemoglobin (HbA1c), ferritin, albumin, and lipids (triglycerides, low density lipoprotein LDL, and high density lipoprotein HDL) were determined after fasting > 3 h (43/65 cases fasting > 12 h).

The non-alcoholic fatty liver disease fibrosis score was calculated as follows Score = −1.675 + 0.037× age (years) + 0.094× body mass index (kg/m^2^) + 1.13× diabetes (yes 1, no 0) + 0.99× AST/ALT ratio − 0.013× platelet (Gpt/l) − 0.66× albumin (g/dl) [37].

Genotyping of PNPLA3 variant p.I148M (rs738409, allele C/G) was performed according to a previously described protocol with some modifications [38]. Briefly, we extracted genomic DNA from peripheral blood leukocytes and performed polymerase chain reaction (PCR) with subsequent melting curve analysis. PNPLA3 genotypes were determined by analytical melting using a pair of fluorescent resonance energy transfer (FRET) hybridization probes complementary to the mutated sequence. Primers and probes were synthesized according to the published nucleotide sequence (GenBank: NG_008631): Forward primer 5′-CTTATGAAGGATCAGGAAAATTAAA-3′, Reverse primer 5′-GGGACAGACCCTGAGGT-3′, Anchor probe 5′-ACCACGCCTCTGAAGGAAGGAGGGATAAG-FL-3′, Sensor probe 5′-LC610-CCACTGTAGAACGGCATGAAGC-PH-3′.

### Statistical analysis

Ordinal and nominal data were collected in a Microsoft® Excel file. Statistical analyses were conducted by using MedCalc 12.7 (MedCalc Software, Belgium) and the R statistical package (Version 2.14.0) and the pROC sub-package for receiver operating characteristics [39]. Clinical and laboratory data were expressed as median and range or mean ± standard deviation (SD), as appropriate. Elastography, CAP and ^1^H-MRS results are presented as boxplots and strip charts.

Fisher's exact test and chi-square test were used to test for association of variables. Nonparametric tests were chosen to compare pseudo-median values of two independent samples (Mann-Whitney U test) or groups (Kruskal-Wallis test) or expected trends (one-sided Jonckheere-Terpstra test). The t-test was used for comparison of mean values of independent samples. For trends in the mean, an ANOVA with polynomial contrasts was performed, where the p-value for the linear term was used after verifying that higher order terms did not contribute significantly. Post-hoc analyses after finding a group effect looked at all combinations of pairs or contiguous pairs if a trend test was performed and corrected for multiple testing using Bonferroni-Holm correction. If such a correction was employed, the corresponding p-value is denoted a “corrected p-value”. Correlations between variables were examined using Pearson's correlation coefficients and the comparison of two sub-groups used the Fisher z-transform [40]. A p-value < 0.05 was considered significant.

The potential influence of biopsy age was investigated by including it both as a continuous and categorial variable in linear/logistic regressions.

Diagnostic performance of the non-invasive methods was evaluated using receiver operating characteristic curves. The probabilities of a true-positive (sensitivity, sens.) and a true-negative (specificity, spec.) were estimated as the proportions in the cohort (i.e. maximum likelihood) and Wilson confidence intervals were constructed. The area under the receiver operating characteristics curve (AUC) was calculated using the trapezoidal rule, where confidence intervals were found according to [41].

### Cut-off calculation

For the ROC curve analysis, cut-off values optimizing the Youden index were calculated for comparison with previous results, some of which implicitly stressed high sensitivity and others high specificity. In addition, we applied published cut-off-values for TE and CAP to our study cohort: For TE, a cut-off of 7.9 kPa has been described for sensitive identification of patients at risk of advanced NAFLD associated fibrosis in a large biopsy controlled cohort [42]. More recently, CAP had a sensitivity > 90% for the detection of hepatic steatosis ≥ S1 and ≥ S2 in patients with chronic liver disease at a cut-off value of 215 and 252 dB/m, respectively [19].

For the CAP procedure an additional cut-off value considering the specific clinical diagnostic requirements was determined by introducing a score to be optimized and estimating the associated confidence interval with bootstrapping procedures.

## Results

### Clinical characteristics of the study cohort

We recruited 53 patients with NAFLD and 17 healthy volunteers. However, three patients had histological and clinical features of concomitant autoimmune or cholestatic liver disease. Two control cases showed signs of liver disease (one with steatotic liver ultrasound pattern, one with elevated aminotransferases) and were excluded. Therefore, 50 patients with NAFLD and 15 healthy controls were included in the final analysis.

Patients with NAFLD were classified by a single expert pathologist according to the histological degree of steatosis: 18, 20, and 12 cases had mild (S1), moderate (S2), and severe (S3) steatosis, respectively [1], [29]. Fibrosis staging revealed F0, F1, F2, F3, and F4 fibrosis in 10, 32, 2, 3, and 3 cases. The associations between CAP, ^1^H-MRS and histology were essentially unaffected by the age of the biopsy (all p-values > 0.3).

Clinical characteristics of the study cohort are presented in table 1. Gender distribution did not differ significantly between the three NAFLD subgroups and only slight differences in age were observed. Time span since liver biopsy did not differ significantly. NAFLD cases with mild steatosis (S1) had a lower prevalence of hepatocellular inflammation (NASH) (n = 4/18 vs. 22/32, p = 0.003), arterial hypertension (n = 4/18 vs. 20/32, p = 0.008), and a trend towards a lower prevalence of diabetes mellitus type 2 (n = 2/18 vs. 12/32, p = 0.056) compared to patients with more advanced steatosis (S2 and S3). Moreover, S2 and S3 patients had a higher frequency of the non-CC *PNPLA3* genotype (n = 22/32 vs. 4/18, p = 0.003) and a trend towards a higher risk profile according to the NAFLD score (intermediate/high risk n = 16/32 vs. 4/18, p = 0.074) (table 1).

**Table 1:** Baseline characteristics of the study cohort.

		Healthy Controls	Patients with NAFLD		
Degree of steatosis	NAS	(S0)	S1	S2	S3
**Anthropometry**					
sex	male/female	6/9	11/7	9/11	5/7
age*	years	38.5 ± 11.8	50.4 ± 12.9	60.0 ± 7.5	54.7 ± 9.1
BMI*	kg/m^2^	22.9 ± 2.4	25.9 ± 4.1	29.0 ± 4.0	33.0 ± 4.9
waist-to-hip*	ratio	0.86 ± 0.13	0.90 ± 0.09	0.96 ± 0.08	0.96 ± 0.06
**Comorbidities**					
diabetes mell. type 2	n	0	2 (11%)	6 (30%)	6 (50%)
arterial hypertension	n	0	4 (22%)	12 (60%)	8 (67%)
**Histology**					
time since biopsy*	months	-	14.8 ± 14.4	13.3 ± 12.9	17.6 ± 14.6
fibrosis	F0	(15)	8	1	1
	F1	-	9	14	9
	F2	-	0	1	1
	F3	-	0	2	1
	F4	-	1	2	0
inflammation	absent/present	(15/0)	14/4	6/14	4/8
**PNPLA3 genotyping**					
rs738409 CC/CG/GG	n	8/6/1	14/4/0	7/10/3	3/8/1
**NAFLD Score**					
low risk	n	14	14	11	5
indeterminate risk	n	1	3	7	7
high risk	n	0	1	2	0
**Laboratory values**					
ALT/ULN*	ratio	0.43 ± 0.09	1.20 ± 1.13	1.32 ± 1.06	1.04 ± 0.31
AST/ULN*	ratio	0.53 ± 0.10	0.89 ± 0.44	1.07 ± 0.63	0.98 ± 0.40
GGT/ULN*	ratio	0.41 ± 0.24	2.51 ± 3.43	2.05 ± 1.82	1.98 ± 2.13
HbA1c*	(%)	4.98 ± 0.21	5.14 ± 0.50	5.56 ± 0.71	6.04 ± 0.96
ferritin/ULN* ^,^ **	ratio	0.50 ± 0.56	0.82 ± 0.70	1.08 ± 0.77	0.88 ± 0.85
triglycerides*	(μmol/l)	0.97 ± 0.49	1.17 ± 0.51	1.77 ± 0.89	2.18 ± 1.19
LDL cholesterol*	(mmol/l)	2.74 ± 0.80	3.48 ± 0.82	3.49 ± 1.08	3.59 ± 1.19
HDL cholesterol*	(mmol/l)	1.90 ± 0.43	1.77 ± 0.62	1.39 ± 0.44	1.41 ± 0.39

* values presented as mean and standard deviation.

** values missing in three individuals (2x S1, 2x S3).

Abbreviations: BMI – body mass index, ULN – upper limit of normal.

### Transient elastography and performance of CAP

Valid and reliable TE and CAP results could be obtained in all control cases (100%) and 46/50 patients with NAFLD (92%). Patients with fibrosis stage F2–4 (n = 8) had higher median TE values (18.0 kPa) compared to NAFLD cases with stage F0–1 and controls (4.8 kPa, n = 53), 95% CI for increase in median [9.0, 22.4] kPa, p<0.0001. The ROC curve analysis for differentiation between F0–1 and F2–4 fibrosis revealed a high accuracy (sensitivity 100% [67.6, 100]%, specificity 98.1% [90.1, 99.9]%, AUC 0.991 [0.971, 1]) at a cut-off value of 8.85 kPa.

**Figure 1:**
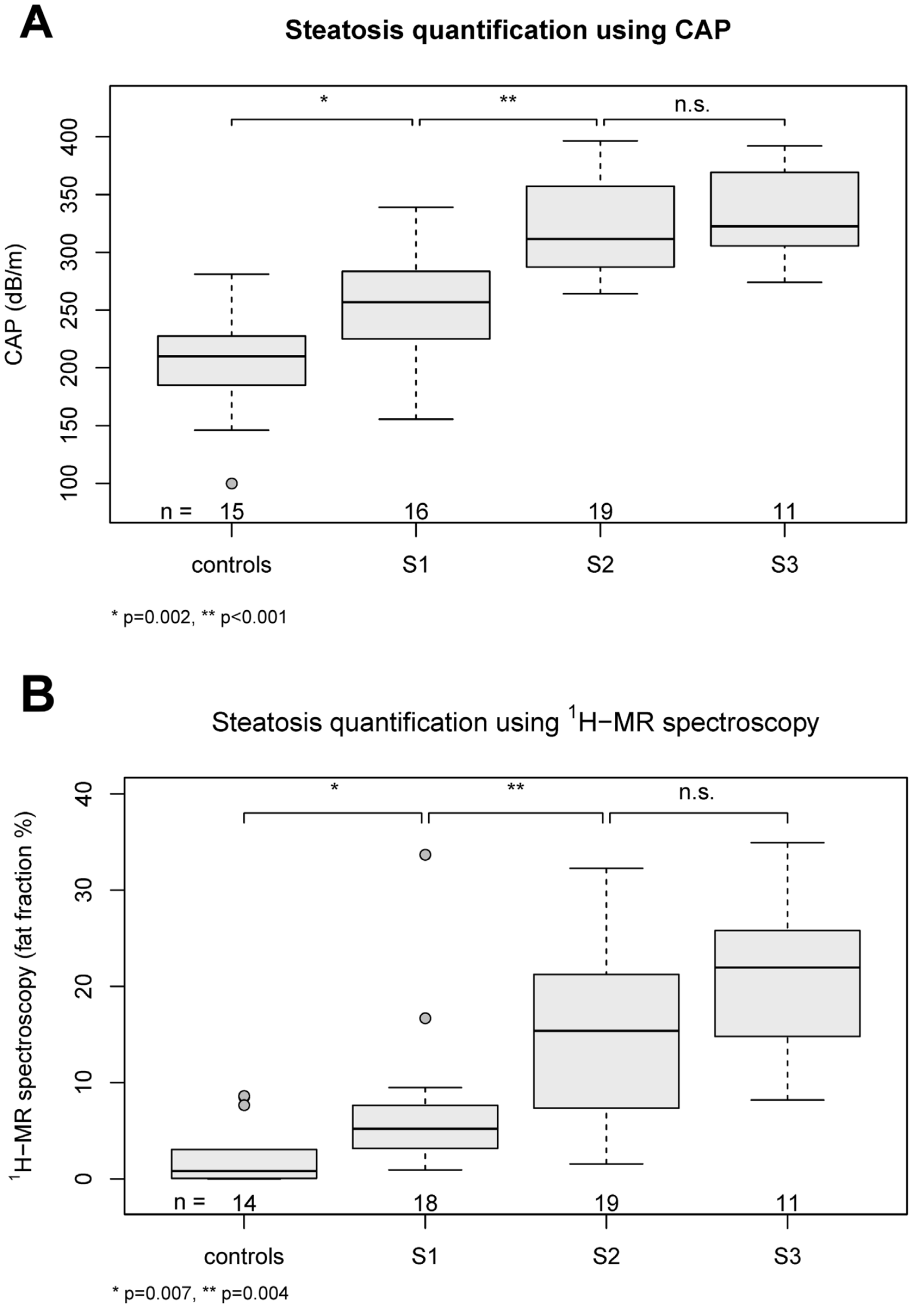
CAP (A) and ^1^H-MRS (B) correlate with hepatic steatosis. CAP and ^1^H-MRS values correlate with the amount of hepatic fat and show a stepwise increase compared to the NAS staging (61 and 62 valid measurements available, respectively).

Median CAP values increased significantly with the degree of hepatic steatosis (p < 0.0001) and differed by an estimated 52 dB/m ([19, 80] dB/m, corrected p = 0.002) between the controls and S1 and by 67 dB/m ([40, 80] dB/m, corrected p = 0.0001) between S1 and S2 (table 2, figure 1A). ROC curve analysis revealed a high accuracy for differentiation between absence vs. any degree of steatosis (S0 vs. S1–3, AUC 0.930 [0.865, 0.996]) and mild vs. moderate/severe (S0–1 vs. S2–3, AUC 0.934 [0.883, 0.994]) hepatic steatosis, whereas the area under the curve for differentiation between S0–2 and S3 steatosis was 0.816 [0.701, 0.932] (table 3).

**Table 2:** Elastography, Controlled attenuation parameter (CAP) and magnetic resonance imaging.

		Healthy Controls	NAFLD Patients – degree of steatosis			p-value
		(S0)	S1	S2	S3	
**Transient Elastography**	valid/all	15/15	16/18	19/20	11/12	0.6
skin-to-liver-capsule distance*	mm	16.3 ± 2.7	19.7 ± 3.7	23.5 ± 6.4	26.2 ± 5.3	p < 0.0001
liver stiffness**	kPa	4.4 [2.3–5.9]	4.8 [1.9–59.3]	5.3 [2.7–70.6]	5.4 [3.5–21.8]	p = 0.003
CAP*	dB/m	201 ± 44	253 ± 43	321 ± 42	335 ± 43	p < 0.0001
**Magnetic resonance imaging**	available (n)	15	18	19	11	-
liver volume*	ml	1346 ± 223	1435 ± 353	1746 ± 394	2067 ± 390	p < 0.0001
subcutaneous fat volume (L3)*	ml	240 ± 116	283 ± 81	318 ± 99	384 ± 109	p = 0.002
visceral fat volume (L3)*	ml	75 ± 106	113 ± 59	194 ± 103	247 ± 137	p < 0.00001
^1^H-MRS (segment VII)**	rel. lipid signal	0.8 [0–8.6]#	5.2 [0.9–33.7]	15.4 [1.6–32.3]	22 [8.2–34.9]	p < 0.00001

* values presented as mean and standard deviation

** values presented as median and range

# available in 14 cases of the control cohort

Abbreviations: MRS – magnetic resonance spectroscopy

**Table 3:** Diagnostic performance for detection of hepatic steatosis at optimal cut-off (optimizing the Youden Index).

		CAP	^1^H-MRS
**S0 (controls) vs. S1–3**	cases (n)#	15/46	14/48
	Sensitivity	93% [80, 100]%	79% [57, 100]%
	Specificity	87% [76, 96]%	88% [77, 96]%
	AUC*	0.93 [0.86, 1.00]	0.88 [0.78, 0.99]
	cut-off	233.5 dB/m	3.12% fat fraction
**S0–1 vs. S2–3**	cases (n)#	31/30	32/30
	Sensitivity	97% [90, 100]%	91% [81, 100]%
	Specificity	81% [64, 94]%	77% [60, 90]%
	AUC*	0.94 [0.88, 0.99]	0.88 [0.79, 0.97]
	cut-off	268.5 dB/m	8.77% fat fraction
**S0–2 vs. S3**	cases (n)#	50/11	51/11
	Sensitivity	82% [55, 100]%	91% [73, 100]
	Specificity	76% [64, 88]%	75% [63, 86]
	AUC*	0.82 [0.70, 0.93]	0.85 [0.75, 0.95]
	cut-off	301.2 dB/m	13.69% fat fraction

# Only patients with valid measurements were considered for this analysis.

* Comparison of CAP and ^1^H-MRS ROC curves did not reveal significant differences of AUC.

### MR imaging and performance of MR spectroscopy

All healthy subjects and 48 patients with NAFLD (96%) underwent MRI examinations. One patient was excluded from MRI due to a contraindication (pacemaker), one patient refused examination because of claustrophobia. In addition, ^1^H-MRS data from one healthy volunteer were lost due to a technical error during data acquisition.

Liver volume and visceral fat volume showed a stepwise increase compared to the degree of hepatic steatosis. A similar trend was observed for the subcutaneous fat volume (post-hoc analyses could verify only that it was significant for S0 vs. S3 however) (table 2).

**Figure 2:**
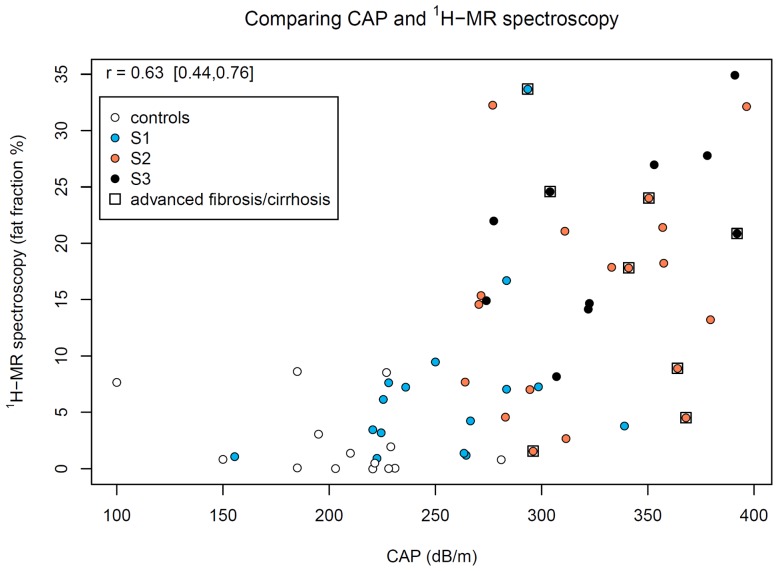
Correlation of CAP and ^1^H-MRS. CAP and ^1^H-MRS achieved only a modest correlation, especially in patients with concomitant fibrosis (labeled with squares). A total of 61 valid measurements were available.

The median hepatic fat fraction measured by ^1^H-MRS also increased significantly with the degree of hepatic steatosis (p<0.001) with an estimated change of 3 percentage points ([1], [6], corrected p = 0.01) between the control group and S1, of 9 percentage points ([2], [14], corrected p = 0.01) between S1 and S2 and an estimated 6 percentage points ([−1, 13], p = 0.1) between S2 and S3 (table 2, figure 2). All degrees (S1–3), moderate/severe (S2–3), and severe (S3) hepatic steatosis were detected with good accuracy (AUC > 0.85, lower end of 95% CI > 0.75) (table 3, figure 1B).

### Comparison of CAP and MR spectroscopy

Correlation of CAP and ^1^H-MRS was analyzed using Pearson's correlation coefficient. Both methods correlated modestly in the total study cohort (n = 58 with available valid CAP and ^1^H-MRS results, r = 0.63 [0.44, 0.76], p<0.001), while analysis of NAFLD cases only resulted in an even weaker correlation (n = 44; r = 0.56, [0.32, 0.74], p<0.001). We therefore further analyzed the influence of liver fibrosis on correlation between CAP and ^1^H-MRS: the correlation in patients with biopsy proven liver fibrosis F2–4 (n = 8, r = −0.2, [−0.8, 0.6]) differed significantly from the correlation for those with no or mild fibrosis (F0–1) (r = 0.7, [0.5, 0.8]), in whom the comparison of these correlations resulted in p<0.0001 (figure 2). The individuals with F2–4 fibrosis showed higher ferritin levels compared to cases with F0–1 fibrosis (median 113%ULN vs. 59%ULN, difference in pseudo-median 70 percentage points [11, 207], p = 0.02).

### Cut-off values for clinical use

**Figure 3:**
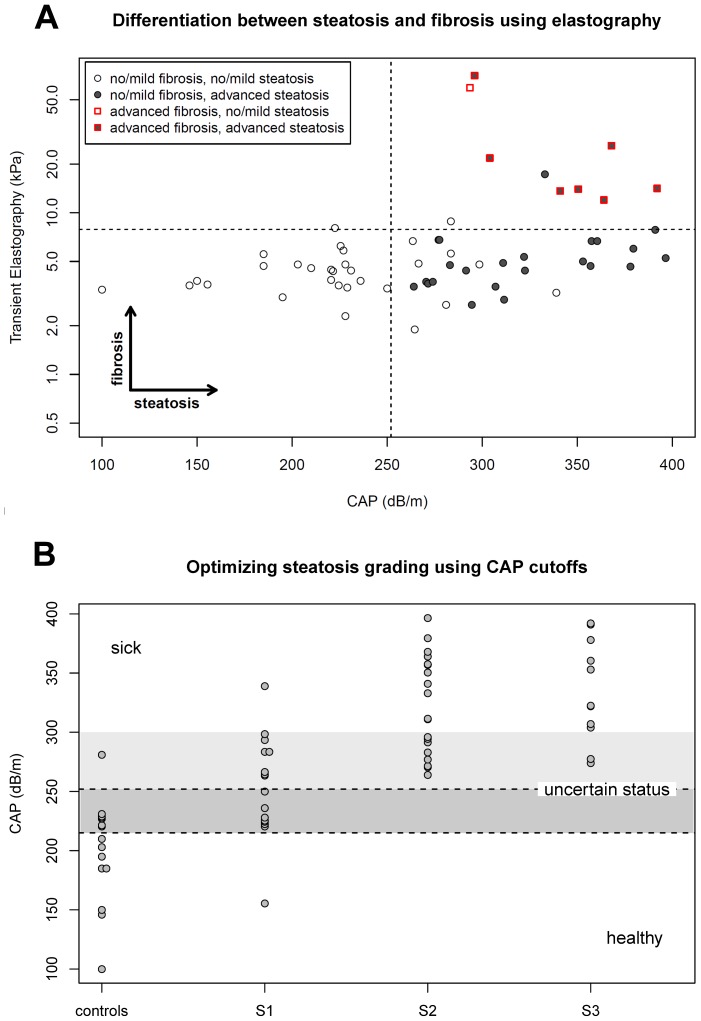
CAP and TE cut-off values for clinical use. Application of published cut-off values for TE (7.9 kPa) and CAP (252 dB/m^2^) results in high sensitivity for detection of distinct fibrosis and steatosis (A) [19], [43]. CAP values between 215 dB/m [19] and 300 dB/m require further diagnostic procedures for differentiation of the degree of steatosis (“grey area”) (B).

Applying a TE cut-off value of 7.9 kPa [42] to our cohort resulted in 100% [68, 100]% sensitivity for the detection of F2–4 fibrosis with a specificity of 94% [85, 98]%. Similarly, a CAP cut-off of 252 dB/m resulted in a 100% [89%, 100%] sensitivity for detecting and S2–3 steatosis, but a specificity of only 71% [53%, 84%] (figure 3A). At the 252 dB/m threshold, steatosis quantification was accurate for controls and patients with steatosis S2 and S3, while 8/16 (50%) of S1 patients with valid CAP results were misclassified. These eight patients had a trend toward a higher BMI (27.0±4.1 vs. 23.6±2.3 kg/m^2^, p = 0.06), NAFLD score (−1.4±1.6 vs. −3.0±1.2, p = 0.04) and skin-to-liver-capsule distances (20.8±3.3 vs. 17.5±2.1, p = 0.03) compared to correctly classified S1 patients. Only one of the misclassified patients had a skin-to-liver-capsule distance above 25 mm (27.2 mm).

In clinical practice diagnostic tests with high specificity are required [43]. This requirement cannot be met with a single cut-off and we thus chose to employ a second, higher one, which will permit us to improve specificity at the price of introducing the category of “unclassified” patients requiring further testing.

We did so by determining the CAP value for the detection of steatosis ≥ S2 that maximizes the score

score  =  (true positives + 0.5 true negatives −3 false positives – false negatives −0.25 unclassified)/(n_patients + 0.5 n_control)

which has the value 1 for a perfect test. While the choice of four coefficients may seem to permit a large amount of arbitrariness, we point out that other methods, such as optimizing a ROC curve or the sensitivity, make similar choices implicitly. They lack however the freedom to provide a weight to each item as appropriate to the diagnosis under scrutiny.

**Figure 4:**
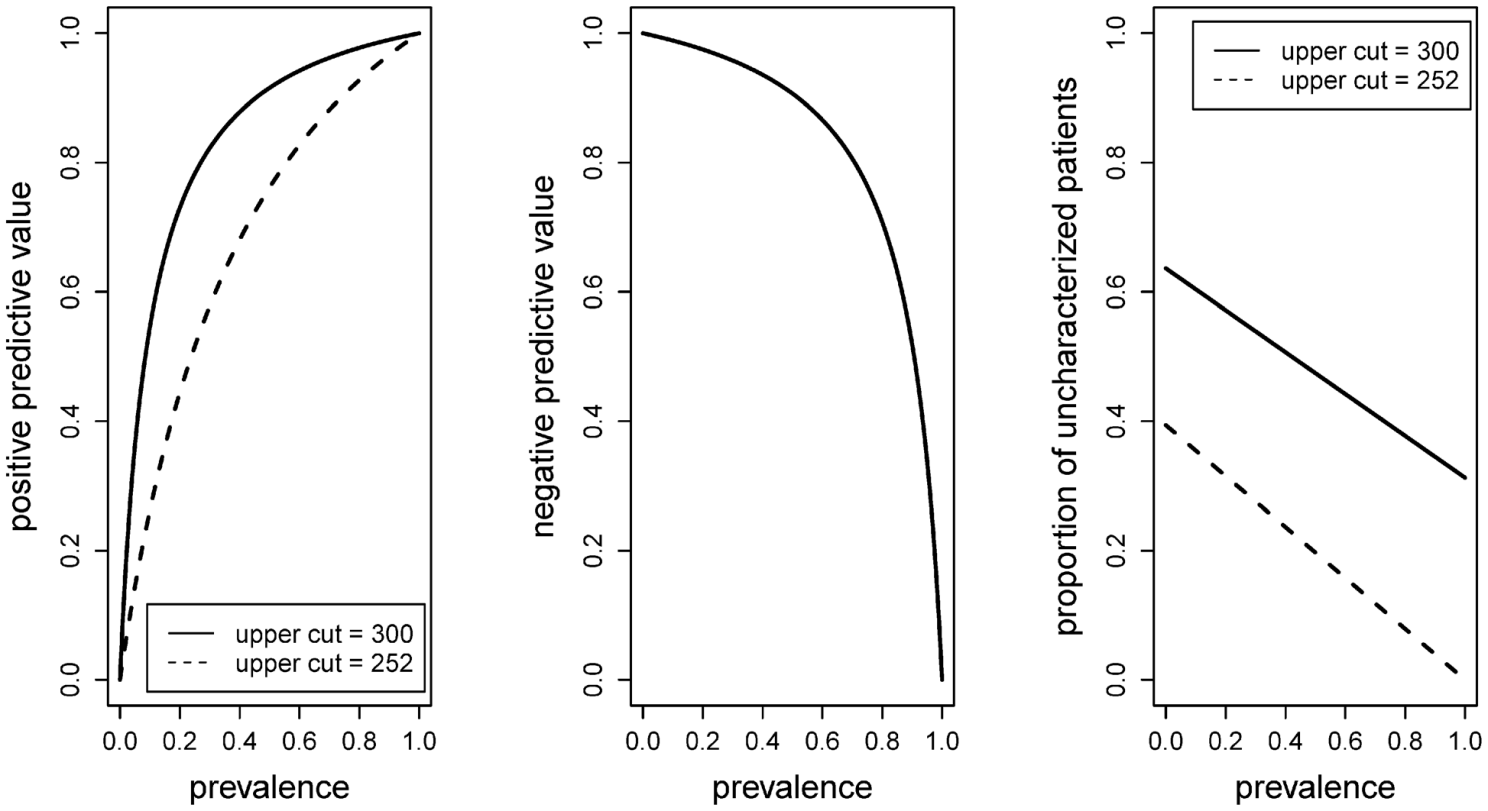
Positive and negative predictive values as well as proportion of uncharacterized cases as they depend upon prevalence. S0–1 patients and controls were classified as healthy and S2–3 as sick. The diagnostic procedure made use of CAP where those with values below 215 dB/m were diagnosed as healthy, those with values above 300 dB/m (or 252 dB/m, dashed lines) were diagnosed as sick and those in between were not diagnosed. A total of 61 valid measurements were available.

This calculation revealed a cut-off of 301 dB/m. Bootstrapping (5000 times repetition) with a random selection of half the data points results in a median cut-off of 294 dB/m with 95% of the values in the interval [257, 345] dB/m. We therefore used a cut-off of 300 dB/m for the detection of S2–3 steatosis to obtain high specificity, while retaining the lower cut of 215 dB/m for the definition of healthy individuals (figure 3B). Applying these cut-offs to the 61 patients/controls with valid CAP measurements results in 20 true positive and one false negative diagnosis, 9 true negative and no false negative cases and 31 who are unclassified and require further testing. The implications regarding positive and negative predictive values and the proportion of unclassified cases can be found in figure 4. There, one can see that predictive values are above 80% for prevalences roughly between 0.25 and 0.7, but with 40 to 60% of the patients remaining uncharacterized.

## Discussion

Non-invasive hepatic fat quantification has a growing importance for the diagnosis and monitoring of hepatic steatosis [15], [16]. To our knowledge, this is the first study that provides a head-to-head comparison of the ultrasound based CAP technology and MR spectroscopy for hepatic steatosis quantification. CAP is calculated from the TE ultrasound signal attenuation and can therefore only be acquired given a successful TE measurement [27]. Thus, limitations of TE – mainly obesity – likewise impair CAP feasibility [27], [31], [44]. In addition, influencing factors on CAP accuracy and quality standards (e.g. exclusion of results with high IQR values) are not yet defined [18], [19]. In our study cohort, although CAP results were only considered when TE fulfilled the commonly accepted quality criteria [19], a high success rate was observed for both healthy individuals (100%) and patients with NAFLD (92%).

1H-MRS techniques expl°it the difference in resonance frequencies between water and fat signals for estimation of hepatic fat concentration and usually have a high feasibility (>90% in our cohort) [28]. Their application is limited by implanted medical devices and claustrophobia, and can also be impaired by severe obesity [45].

CAP and ^1^H-MRS each correlated fairly well with biopsy proven steatosis and significantly differentiated between S0, S1 and S2 patients (figure 1). However, a significant difference could not be detected in subjects with advanced disease (S2–S3), which is in line with the CAP data of Myers et al. [18] and de Ledinghen et al. [19] and may limit adequate monitoring of steatosis changes in these patients.

In the head-to-head comparison between both methods, CAP and ^1^H-MRS achieved only a modest correlation, especially in patients with concomitant fibrosis (figure 2). In these individuals increased serum ferritin levels suggest NASH associated hepatic iron deposition [46] which can interfere with MR-based steatosis assessment and thus may have contributed to imprecise liver fat quantification [47], [48]. Correlation may be further impaired by anthropometrical characteristics like high BMI or large skin to liver capsule distance, which can affect accuracy of transient elastography and hence CAP [22], [49], [50] and cannot be assessed without the additional use of ultrasound.

Anthropometrically related limitations of the TE and CAP technology may be overcome by further development of the TE XL probe. At present, CAP is only available for the M probe of the transient elastography system which reduces feasibility and reliability in patients with BMI values > 28 kg/m^2^ and high skin-to-liver capsule distances [51]. Implementation of CAP in the TE XL probe, which is designed for liver stiffness assessment in obese patients, may overcome this limitation in the future [27], [51].

Considering non-invasive methods as an alternative to liver biopsy, cut-off values for steatosis grading are required. Our ^1^H-MRS cut-off value for the detection of any steatosis grade (3.12% fat fraction) corresponds to data from Szczepaniak et al. who determined a hepatic fat fraction signal of 5.56% as upper 95th percentile in healthy individuals [23]. However, further biopsy controlled studies applying ^1^H-MRS did not provide cut-off values thus rendering a comparison difficult [17], [52], [53].

Our CAP cut-off values for different grades of hepatic steatosis are in line with data from previous biopsy controlled studies evaluating CAP in various chronic liver diseases [17]–[21]. To date, CAP and ^1^H-MRS results show a distinct overlap between NAFLD subgroups (figure 1) which affects correct classification in a considerable proportion of patients: depending on the applied cut-off value and the estimated prevalence of steatosis in the population of interest, CAP correctly classifies 50% of individuals with absent/mild (S0–1) or moderate/severe (S2–3) steatosis (figure 3b, figure 4). The introduction of two cut-offs is important for dealing with this issue and improving the predictive value of the technique. We point out that one cannot expect, or even strive for very high predictive values so long as the gold standard itself contains a considerable amount of error. As described above, this is the case with biopsies, meaning that the “correct” diagnosis itself may be erroneous in a considerable fraction of cases [11], [12].

However, care for patients with NAFLD in clinical practice requires not only grading of hepatic steatosis, but precise cut-off values for identification of “healthy” and “sick” individuals. Therefore, a clear diagnostic and therapeutic concept is necessary when non-invasive methods of steatosis grading are implemented in clinical routine or as diagnostic tools in clinical studies with special regard to patients assigned to the “grey area”: Careful patient selection, consideration of interfering factors (e.g. body mass index, skin to liver capsule distance, fibrosis) and combination of different methods may improve the diagnostic accuracy of non-invasive liver assessment and underline its potential as a guidance to liver biopsy [54]. Longitudinal studies are required to define the value of CAP as a monitoring tool for hepatic steatosis in interventional studies.

In addition to the steatosis evaluation with the CAP technology, transient elastography could simultaneously diagnose hepatic fibrosis. Applying the cut-off value of 7.9 kPa, which has been proposed for patients with NAFLD with F3-4 fibrosis by Wong et al. [42], fibrosis could be detected in all individuals at risk for disease progression (cases with F2–F4 fibrosis) in our cohort (figure 3A). Differences in the classification of F2 patients in our study compared to Wong et al. may be related to the baseline characteristics of the study cohorts, as Wong et al. included 48% individuals with Chinese ethnicity [42] and the low case numbers. Thus, transient elastography with the M probe and simultaneous CAP measurement are promising tools to non-invasively characterize major histopathological aspects of patients with NAFLD. In the future, these methods have to prove recent data for the NAFLD fibrosis score which could show that non-invasive characterization of NAFLD better predicts long-term outcome than histology [55].

Our study has some limitations:

- We used liver histology as a reference standard for steatosis grading and fibrosis staging. Histology classifies steatosis according to the number of affected hepatocytes without assessing the hepatic triglyceride concentration [29]. This limits its comparability with hepatic fat fraction measurement by ^1^H-MRS and ultrasound signal attenuation calculation by CAP. In this regard, further biopsy controlled longitudinal studies are required to determine whether the proportion of affected hepatocytes, the type of steatosis (micro- or macrovesicular), or the hepatic lipid concentration are the best marker for disease severity and risk of progression.
- We cannot exclude alteration of histological NAFLD features during the time interval from biopsy to study inclusion. Although unlikely for simple steatosis [56], significant disease progression may have contributed to the finding of narrow differences of CAP and ^1^H-MRS results in patients with S2 and S3 steatosis. Our analyses could not detect any such effect however. In addition, liver biopsies were not performed as part of our study examinations for ethical reasons as this invasive procedure would not have influenced the clinical management in our NAFLD patients.
- Our results were acquired in a NAFLD population with a limited prevalence of advanced liver damage (16% of cases with ≥F2 fibrosis). Therefore, further studies are required to investigate the correlation of CAP and ^1^H-MRS in cohorts with advanced NAFLD stages.

In conclusion, our pilot data suggest a comparable diagnostic accuracy of CAP and ^1^H-MRS for non-invasive characterization of hepatic steatosis. Together with the simultaneous fibrosis assessment by transient elastography, it represents a fast and easy to use method to characterize patients with NAFLD non-invasively. Considering the increasing NAFLD prevalence, its growing medical impact, and the need for easy, repetitive, and reliable diagnostic tools, it is encouraging that CAP can already correctly classify 50% of individuals with values <215 dB/m excluding hepatic steatosis and results >300 dB/m identifying > 33% steatosis.
